# Significance and Frequency of Incidental Fortuitous Clinical Identifications of Hypertrophic Cardiomyopathy

**DOI:** 10.1016/j.jacadv.2024.100948

**Published:** 2024-04-25

**Authors:** Sonu Abraham, Ethan J. Rowin, Anu Mariam Saji, Jonathan S. Silver, Prem S. Shekar, Martin S. Maron, Barry J. Maron

**Affiliations:** HCM Center, Lahey Hospital and Medical Center, Burlington, Massachusetts, USA

Hypertrophic cardiomyopathy (HCM) is a heterogeneous often inherited cardiac disease which relies on echocardiography and/or cardiac magnetic resonance imaging for definitive diagnosis.^1^^,^^2^ Notably, HCM is now a contemporary treatable disease compatible with normal longevity, underscoring the importance of prompt diagnosis in the medical community.

HCM may be suspected in a multitude of clinical scenarios that can lead to diagnostic testing, that is, typically, onset of cardiac symptoms or family screening.^3^ Alternatively, HCM diagnosis may arise from accidental and unexpected clinical circumstances.^3^ Therefore, we have studied a large patient cohort prospectively to assess the frequency and significance of HCM diagnoses in a variety of clinical situations.

The HCM Center at Lahey Hospital and Medical Center was established on April 14, 2022. Over the next year, 917 consecutive patients were evaluated for HCM as outpatients. Study patients were referred to our HCM center for diagnostic confirmation; family screening; cardioverter-defibrillator implantation (ICDs); heart failure treatment with drugs, surgical myectomy/percutaneous alcohol ablation, or management of atrial fibrillation. Details of clinical circumstances surrounding the HCM diagnoses were assessed. Follow-up was from initial clinic visit (and study entry) to September 1, 2023.

Of the 917 study patients, age at initial evaluation was 58.7 years. Patients were initially suspected of cardiac disease for a variety of reasons: most commonly symptoms typical of HCM (eg, exertional dyspnea and syncope) in 580 or family screening for HCM in 92.

In 235 patients (25%), HCM was suspected from clinical findings or observations considered “incidental,” that is, made unexpectedly, often in the course of routine primary care or general medicine evaluations, and subsequently confirmed by diagnostic imaging.

Of 235 incidentally identified patients, age at initial evaluation was 58.4 years; 71% were male (Figure 1). Left ventricular (LV) wall thickness was 17.9 mm and 114 (48%) had LV outflow gradients ≥30 mm Hg either at rest or with Valsalva. Most (n = 215, 91%) had no or mild symptoms (New York Heart Association functional classes I/II).

**Figure 1:**
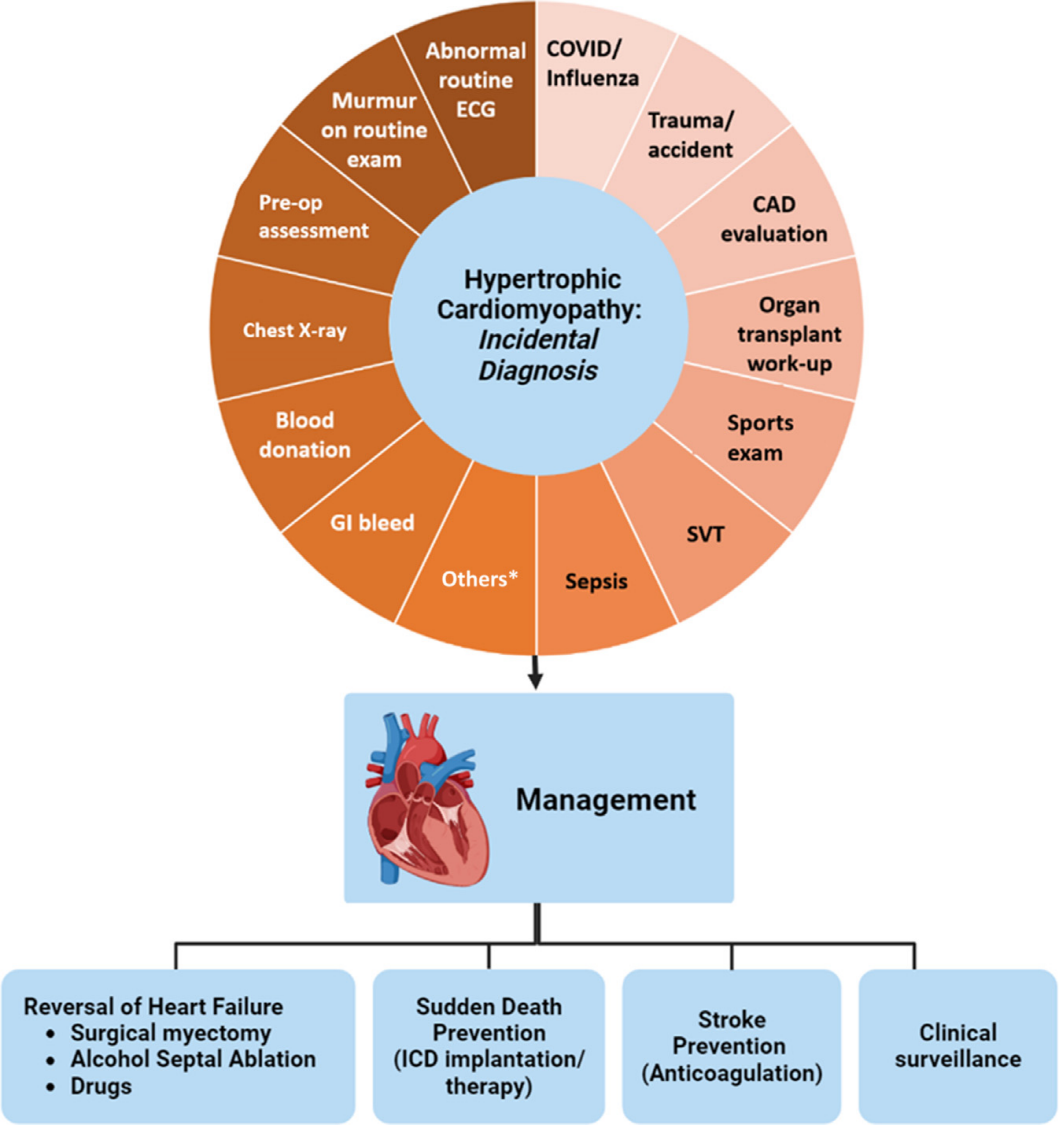
**Triggers for HCM Diagnosis in “Real-World” Settings Based on Incidental Clinical Findings** ∗Evaluation for systemic hypertension; pericarditis; pulmonary arterial hypertension; muscular/dystrophy; seizure disorder; valvular heart disease; differential diagnosis of “athlete’s heart”. CAD = coronary artery disease; GI = gastrointestinal; HCM = hypertrophic cardiomyopathy; SVT = supraventricular tachycardia.

Incidental clinical factors leading to suspicion and ultimately HCM diagnosis were numerous and varied. Most common were unexpected recognition of a precordial systolic murmur (n = 77; 68% of patients with outflow obstruction), or 12-lead electrocardiogram abnormalities (n = 44), most commonly deep T-wave inversion (3-15 mm) in lateral precordial leads (V_1_-V_6_), present in 45% of nonobstructive patients with apical hypertrophy or LV aneurysm. Together, murmurs and electrocardiogram abnormalities accounted for 51% of incidentally diagnosed patients.

Other incidental HCM diagnoses followed evaluations for cardiovascular but non-HCM diseases, for example: acute myocardial infarction (n = 21); non-cardioembolic stroke (n = 11); aortic valve disease/pericardial disease (n = 7); and systemic hypertension (n = 5).

Evaluations for non-cardiovascular diseases also led to incidental HCM diagnoses: noncardiac surgery (n = 21); sepsis or gastrointestinal bleeding (n = 13); COVID/influenza treatment (n = 8); insurance or licensure assessments and military or preparticipation sports evaluations (n = 13); blood donations or noncardiac organ transplants (n = 6); and trauma such as car accidents (n = 4).

In 94 of 235 incidentally identified HCM patients (40%), this diagnosis led to beneficial treatment strategies. Thirty patients with LV outflow obstruction developed progressive heart failure symptoms requiring invasive septal reduction intervention (septal myectomy [n = 26] and alcohol ablation [n = 4]).^4^ Improvement from New York Heart Association functional class III to I-II was associated with reduction in outflow gradient from 67 to <5 mm Hg.

Notably, 33 (14%) of the incidentally diagnosed patients were considered at high sudden death risk and received primary prevention implantable cardioverter-defibrillators (ICDs), based on ≥1 major American Heart Association/American College of Cardiology risk markers.^5^ Notably, 3 of these 33 ICD patients (9%) experienced appropriate device therapy terminating ventricular tachycardia/ventricular fibrillation, at 42, 52, 68 years of age (2 were males).

In addition, after incidental diagnosis, 31 patients developed paroxysmal or persistent atrial fibrillation/flutter. Each were treated with anticoagulants (direct novel agents in 30) as well as anti-arrhythmic drugs (n = 10), catheter ablation (n-8), and Cox-Maze IV surgical ablation (n = 1). There have been no embolic stroke events, except in 1 patient who self-terminated anticoagulation. Therefore, 40% of the fortuitously diagnosed HCM patients have achieved effective guideline-recommended treatment^1^; all 235 patients have survived to the end of follow-up.

Unexpected fortuitous diagnoses have become increasingly common in medicine.^3^ While most HCM diagnoses in our cohort were triggered by cardiac symptoms, we nevertheless identified a significant minority (about 25%) that were due to a variety of clinical scenarios, usually within primary care or general medical settings. Most commonly, these were unexpected precordial murmurs or electrocardiogram abnormalities, but also less frequent circumstances such as insurance or licensure assessments, and military and preparticipation sports examinations.

The 235 incidentally identified patients were characterized by: an average age of 58 years; predominantly male (71%); moderate LV wall thickness (18 mm), about one-half with LV outflow obstruction; and >90% with no or mild symptoms.

Most importantly, incidental HCM diagnoses often proved clinically relevant in a variety of ways: prevention of sudden death, reversal of heart failure, and prevention of embolic stroke. The 30 patients with progressive heart failure due to outflow obstruction required septal reduction (eg, surgical myectomy),^4^ resulting in symptom relief, with zero operative mortality. Thirty-three high-risk patients were implanted prophylactically with ICDs,^5^ with almost 10% experiencing device therapy terminating VT/VF, thereby altering their clinical course and preventing sudden death.^1^^,^^5^

In conclusion, incidental HCM diagnosis proved common in a consecutive cohort (ie, 25%), and leading to effective treatment strategies in 40%. These observations underscore the importance of vigilance and high index of suspicion for HCM and the need for prompt diagnosis, given that it has become a contemporary treatable disease compatible with normal or extended longevity as well as good quality of life.

The present analysis also identified a window into a vast underdiagnosed (and sometimes undertreated) HCM subpopulation, which probably includes many patients unaware of the effective management options for which they are eligible.

## References

[1] Ommen S.R., Mital S., Burke M.A. (2020). 2020 AHA/ACC guidelines for the diagnosis and treatment of patients with hypertrophic cardiomyopathy. J Am Coll Cardiol.

[2] Maron B.J., Desai M.Y., Nishimura R.A. (2022). Diagnosis and evaluation of hypertrophic cardiomyopathy. JACC state-of-the-art review. J Am Coll Cardiol.

[3] Efthimiadis G.K., Parcharidou D., Pagourelias E.D. (2010). Prevalence and clinical outcomes of incidentally diagnosed hypertrophic cardiomyopathy. Am J Cardiol.

[4] Maron B.J., Dearani J.A., Smedira N.G. (2022). Ventricular septal myectomy for obstructive hypertrophic cardiomyopathy (analysis spanning 60 years of practice) AJC Expert Panel. Am J Cardiol.

[5] Maron B.J., Rowin E.J., Maron M.S. (2021). Evolution of risk stratification and sudden death prevention in hypertrophic cardiomyopathy: twenty years with the implantable cardioverter-defibrillator. Heart Rhythm.

